# University students' perceptions and factors contributing to obesity and overweigh in Southern of Morocco

**DOI:** 10.4314/ahs.v21i2.56

**Published:** 2021-06

**Authors:** Mohamed Boukrim, Majdouline Obtel, Laila Lahlou, Rachid Razine

**Affiliations:** 1 Laboratoire of Biostatistics, clinical Research and Epidemiology- Faculty of Medicine and Pharmacy. Mohamed V University, RABAT, Morocco; 2 Social Medicine Laboratory (Public Health, Hygiene and Preventive Medicine) - Faculty of Medicine and Pharmacy. Mohamed V University, RABAT, Morocco; 3 Faculty of Medicine and Pharmacy, Ibn Zohr University, AGADIR, Morocco

**Keywords:** Weight load, obesity, overweight, perception, female students, higher education, Morocco

## Abstract

**Background:**

Weight load is a cosmopolitan health problem. In Morocco, women are the most affected by the phenomenon since obesity has risen from 26.8% to 29.0%.

**Objective:**

To determine the prevalence of weight load and associated factors among female students in higher education.

**Methods:**

Data were collected by a questionnaire. Anthropometric measurements were made using a scale and a wall-mounted scale. Data were analysed by the statistical software SPSS version 13.0. Quantitative variables were described in mean ± standard deviation. Factors associated with obesity were determined by binary logistic regression.

**Results:**

About two-thirds of the students had a normal weight, 21% were overweight and 3% were obese. In addition, 58% of students were physically inactive and 77% ate cake and fast food. In addition, 63% of students were dissatisfied with their weight. A significant relationship is found between level of primary education, type of establishment, cake and fast food consumption and weight load.

**Conclusion:**

The results revealed that 24% of participants were weight load and had behavioral risk factors such as a sedentary lifestyle and an unbalanced diet, which requires the promotion of a healthy lifestyle among these students as well as psychological support for those dissatisfied with their body image.

## Introduction

Overweight and obesity are defined as an abnormal or excessive accumulation of body fat that can be detrimental to health^1^. They have consequences for physical, psychological and social well-being. The health repercussions of being overweight are well known. It is a risk factor for type 2 diabetes, cardiovascular disease, hypertension, osteoarthritis and certain forms of cancer^2^. Since 1975, the number of obesity cases worldwide has almost tripled. In 2016, 39% of adults aged 18 years and older were overweight and 13% were obese. Most of the world's population lives in countries where overweight and obesity kill more people than underweight^3^. In general, obesity is more prevalent among women than men. Approximately 30-82% of women in the Arab world are overweight and obese compared to 25–70% of men^4^.

Weight load is a cosmopolitan phenomenon. It affects all countries, developed as well as developing. Studies on obesity in Eastern Mediterranean countries, showed that women in Bahrain and Egypt were more likely to be overweight or obese followed by women in Jordan, Syria and Oman^4^. In the Middle East, adults were reported to have the second highest body mass index in the world after those in North America^5^.

In Morocco, this prevalence has evolved between 2011 and 2018 from 50.8% to 53% in the general population, while obesity has increased from 17.9% to 20%. Women are the most affected by this phenomenon. In fact, there has been an increase in the weight load from 61.5% to 63.4%. Obesity increased from 26.8% to 29%^6^,^7^.

Several factors were blamed for the scourge, such as lack of exercise, disturbed sleep, stress, ethnicity, gender, parental education, and family income^8^. Not to mention the influence of the media, family and peers on body satisfaction, with an overall predominance of the effects of media images on body image perception^9^ often leading to the adoption of aspects of westernization^10^. Indeed, recent studies in the Eastern Mediterranean region have revealed westernized visions of ideal body image where the slimmer body image is considered to be better^11^.

Most studies in Morocco have focused on women and children in school, and there has been a lack of studies of the phenomenon among young adults in higher education institutions. The objective of our study was to study the prevalence of weight load and associated factors among young female students in public higher education institutions.

## Methods

This is a cross-sectional observational study conducted in two public institutions of higher education located in the Province of Agadir, capital of the Souss region in Morocco.

These institutions welcome in addition to the students of this region, those of the 3 southern regions. They are the Higher Institute of Nursing and Health Techniques (HINHT) and the National School of Commerce and Management (NSCM). The data collection took place from April to May 2018.

The students were recruited from two institutions that were selected on the basis of the following criteria: accessibility to our field team, presence of a sufficient number of students from neighboring regions and whose officials agreed to the conduct of the study.

The study concerns 200 non-pregnant Moroccan female students over the age of 18. The students had to meet the inclusion criteria and give their consent to participate in the survey. To calculate the sample size, we used the formula:

n = t^2^ × p × (1-p) / m^2^

Where t is the 95% confidence level, p is the estimated prevalence of the obese population, and m is the margin of error (set at 5%).

The prevalence of obesity is estimated at 15% in the same institution^12^. Thus, 195 subjects were considered necessary for inclusion to obtain statistically significant results. The total number of student was 200.

Data Collection was done through a self-administered questionnaire on sociodemographic data (age, level of education of parents, marital status, parents' monthly income), sedentary lifestyle, diet and body image perception. The study was conducted with the free and informed consent of the participants and with respect for the anonymity and the confidentiality of the information.

The anthropometric parameters (weight and height) of the students were measured using the mechanical personal scale marketed name SECA, calibrated in kilograms and a wall scale graduated in centimeters. The measurement of weight (in kg) was carried out on participants with no shoes and in light clothing. Height (in meters) was measured in subjects with their feet flat on the floor, their backs, buttocks and heels pressed against the vertical board of the scale and their heads placed in a horizontal position so that the line of vision was perpendicular to the body.

The body mass index (BMI) was calculated by the following formula: BMI (kg/m^2^) = weight (in kg)/height2 (in m^2^). BMI is recognized as international criteria for assessing corpulence. According to the thresholds adopted by the World Health Organization: underweight is defined as a BMI equal to or greater than 18.5 kg/m^2^, overweight is defined as a BMI equal to or greater than 25 kg/m^2^ and obesity as a BMI equal to or greater than 30 kg/m^2^.

The students' perception of body image was measured by the Silhouette Rating Scale13 or Stunkard Figurines, consisting of a series of nine figures (or silhouettes) representing, from the front, the female body from the leanest to the largest. Each student is invited to indicate which one corresponds best to her (perceived body) and which one corresponds to what she would like to be (ideal body).

Data were analysed using the statistical data processing software SPSS version 13.0. Qualitative variables were described in numbers and percentages and then compared by chi-square and Fisher exact tests considering the conditions of application of each one. Quantitative variables were described in mean ± standard deviation. The prevalence of obesity/overweight was calculated by relating the number of overweight and obese students to the total number of students.

Factors associated with obesity/overweight were determined by binary logistic regression to calculate the odds ratios (OR) and a 95% confidence interval (CI). In all the analyses, P ≤ 0.05 was considered statistically significant.

## Results

The socioeconomic characteristics and the anthropometric parameters of participants are presented in Table 1. The distribution by ethnicity shows that 48.5% and 44% were of Amazigh and Arab ethnicity respectively. More than 52.5% of the parents had a monthly income of at least 7500 Moroccan Dirham (MAD).

**Table 1:** Distribution of female students according to socio-demographic characteristics and body mass index (n=200)

Variables	Mean age ± standard deviation	N	%
**Age**	19,65 ±1.22	-	-
**BMI**	22.20 ±3.525 Kg/m^2^	-	-
**Poids**	58.01±8.86 Kg	-	-
**Taille**	1.62 m ±5.961 cm	-	-
**Level of study**			
1st year		86	43,00
2nd year		59	29,50
3rd year		55	27,50
**Ethnicity**			
Amazigh		97	48,50
Arab		88	44,00
Saharian		15	7,50
**Marital status**			
Single		197	98,50
Married		02	1,00
Divorced		01	0,50
**Education level of parents**			
Illiterate		34	17,00
Primary		41	20,50
Secondary		35	17,50
Academic		90	45,00
**Parents' monthly**			
**income (Moroccan Dirham)**		17	8,50
Less than 2500		54	27,00
2500–5000		34	17,00
5000–7500		42	21,00
7500–10000		53	26,50
More than 10000			
**Body mass index (BMI) in Kg/m2**		32	16.00
Less than 18.5		120	60,00
[18.5–25[		42	21,00
[25–30[		06	3,00
[30–35[			

The mean age was 19.64 ±1.22 years, with a mean weight of 58.01±8.86 Kg kg and an average height of 1.618 m ±5.961 cm. For body mass index (BMI) average was 22.20 ± 3.525 kg/m^2^.

The prevalence of obesity and overweight in our study was 21% and 3% respectively.

Table 2 shows the anthropometric parameters of female student by age strata. The results indicate that the mean of body mass index increased with age up to 22 years.

**Table 2:** Anthropometric parameters of participants by age strata

	Less than 18 years	18–19	20–22	More than 22 years
BMI (kg/m2)	22,11±3.44	22,17±3.84	22,37±2,77	21,89±4,25
Weight (kg)	58,70±8.49	57,64±9.29	58,43±8,10	55,33±12,50
Height (m)	1,63± 0,05.59 cm	1,61± 6.012 cm	1,616±6.23 cm	1,59±,02.89 cm

### Perception and satisfaction with weight

Almost two-thirds (126, 63%) of the students were dissatisfied with their body weight and 65.5% didn't want to gain weight. The reasons given for this refusal were reading magazines and the media, family and friends with proportions of 24%, 23% and 20% respectively.

As for perceptions of obesity and overweight, more than three-quarters considered obesity as a disease and the rest as normal or a sign of beauty.

### Physical activity and sedentary lifestyle

The study revealed that more than half of the female students (58%) were not physically active. Of those students who were active, only 34% were active daily or often. As for the time spent in front of a television screen, 47% of the population studied spent more than 2 hours a day in front of it. Concerning eating cake and fast food, 29.5% of the students said they did it daily (table 3).

**Table 3:** Weight load by student's perceptions and behaviors (n=200)

Variables	N	%	% BMI> 25Kg/m^2^
Satisfaction with your weight*			
No	125	62.50	32
Yes	75	37.50	09.30
**Desire for gaining weight**			
No	131	65.5	34.40
Yes	69	33.50	2.90
**Practice of physical activity**			
No	115	57.50	26.1
Yes	85	42.50	20
**Frequency of physical activity**			
Daily	14	7	0
Often	54	27	24.1
Rarely/never	132	66	25
**Perception of obesity and overweight**			
Normal	42	21	14.3
Disease	158	79	25.9
**TV watching frequency**			
Less than 2 hours a day	107	53.5	20.6
2:00 a.m.-4:00 a day.	62	31	24.2
More than 4 hours a day	31	15.5	32.3
**Frequency of consumption of sugar/fast-food**			
Always	59	29.5	37.3
Often	95	47.5	17.9
Rarely	46	23	17.4

For the perception of body image, three quarters (75.5%) of the students had chosen the image that really represented them. In fact, 74.16% of students with normal BMIs chose figures 3 and 4, which represented normal body images and almost all 91.50% of overweight or obese students considered figures 5 to 9 to be their current body images.

**Figure 1:**
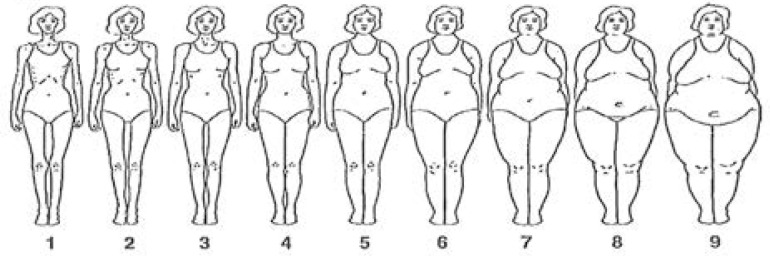
Stunkard Figurines representing the female body from the leanest to the largest

As for the perception of the ideal body, 91% of normal-weight and 81% of overweight students chose Figures 3 and 4 as the ideal body image. However, 40% and 20% of the obese students selected images 5 and 6 corresponding to overweight and obesity respectively (Figure 1 and Table 4).

**Table 4:** Proportion of students as a function of selected figures

Weight load	Number	Silhouette	Figure Number								
			1	2	3	4	5	6	7	8	9
Normal weight	**120**	Current silhouette	3.33	7.5	35.83	38.33	13.33	0.83	0.83	0.0	0.0
		Ideal Silhouette	0.83	3.33	34.16	56.66	5.0	0.0	0.0	0.0	0.0
Overweight	**42**	Current silhouette	0.0	2.4	2.4	4.76	42.85	26.19	14.28	0.14	0.0
		Ideal Silhouette	0.0	2.38	16.66	64.28	16.66	0.0	0.0	0.0	0.0
Obesity	**5**	Current silhouette	0.0	0.0	0.0	0.0	20.0	20.0	0.0	40.0	20.0
		Ideal Silhouette	0.0	0.0	0.0	40.0	40.0	20.0	0.0	0.0	0.0

BMI = body mass index

The univariate logistic analysis revealed that the sugar intake, parental primary education and study institution were significantly associated with weight load (Table 5).

**Table 5:** Factors associated with the weight load among female students in higher education in southern Morocco in univariate analysis (n=200)

Variables	OR	IC à 95%	p	% BMI> 25Kg/m^2^
**Age**	0.97	[0.71–1.21]	0.58	
**Education level**				
1st year	**1**			19.8
2nd year	1.26	[0.57–2.81]	0.57	23.7
3rd year	1.66	[0.76–3.66]	0.20	29.1
**Ethnicity:**				
Amazigh	**1**			20.6
Arab and Sahrian	1.37	[0.71–2.64]	0.35	26.20
**Study institution**				
NSCG	**1**			17.0
HINHT	2.09	[1.07–4.11]	0.032	30.0
**Education level of parents:**				
Illiterate	1.04	[0.39–2.76]	0.94	20.6
Primary	2.31	[1.02–5.23]	0.04	36.6
Secondary	1.00	[0.38–2.65]	1.00	20.0
University	**1**			20.0
**Parental income (Moroccan Dirham)**				
Less than 5000	1.27	[0.61–2.64]	0.51	25.4
5000 to 7500[	1.35	[0.54–3.35]	0.52	26.5
Higher than 7500	**1**			21.1
**TV watching duration**				
Less than 2h	**1**			20.6
2h to 4h	0.54	[0.22–1.32]	0.40	24.2
Higher than 4h	0.67	[0.26–1.73]	0.41	32.3
**Physical exercises**				
No	0.71	[0.36–1.39]	0.32	26.1
Yes	**1**			20.0
**Sugar and fast food using**				
Always	2.82	[1.12–7.14]	0.03	37.3
Often	1.03	[0.41–2.61]	0.94	17.9
Rarely	**1**			17.4

BMI = body mass index IC: Confidence interval OR: Odds Ratio

HINHT: Higher Institute of Nursing and Health Techniques

NSCM : National School of Commerce and Management.

## Discussion

This survey investigated the prevalence of overweight and obesity and associated factors among female students at two institutions of higher education. In this study, almost a quarter of the female students were overweight. In Marrakech 15.7% of female medical students were overweight and 1.3% obese^12^. In other study conducted in the School of Medicine and Health Science of the University of Development Studies in Ghana, obesity was 4.5% among female students^14^. At the Universities of Douala and Nangui Abrogoua in Côte d'Ivoire similar results for overweight (26.44%) were found and for obesity the prevalence was 23%^15^,^16^. At the University of Assuit in Egypt, a study revealed that the prevalence of overweight is 24.6% 11 while it is 33.6% among adolescent girls in Dubai in the United Arab Emirates, 20.5% of whom were obese^10^. The observed differences could be explained by the size of the samples studied, the context of the study, the eating habits and the change in perceptions of overweight and obesity.

Despite the low prevalence of obesity recorded and the low number of female students of Saharawi ethnicity, data analysis also showed that among female students of Saharawi ethnicity, the prevalence of obesity (6.66%) was three times higher than among students of other ethnicities (2.27% among Arabs and 2.06% among Amazigh). These results confirmed those of the study conducted among Sahrawi women in Laayoune^17^. However, there was no significant difference between BMI (obesity and overweight) and ethnic origin.

Univariate logistic regression analysis showed a significant relationship between parents' primary education level and weight load (OR=2.31 IC95% = [1.02-5.23]). Parental income is not associated with the occurrence of obesity/overweight among female students. These results contradict those of other studies^16^,^18^. This may be explained by the nature of the population studied -by these studies- which is composed of adult, non-student women with varying levels of education.

Several other factors conditioned the development of obesity and overweight. Among the students who practiced physical exercise, 58.8% were of normal weight, unlike the students who did not (58%), who were 26% overweight. Similar results among medical students revealed that among students who practiced sports, 88.8% were of normal weight and 22.8% of the others were overweight and obese^12^. However, no association was found between sport practice and obesity/overweight. Studies have shown that eating high-calorie, low-nutrient foods promote overweight and obesity^8^. As a result, the trend towards fast food consumption, especially among young people, may increase energy intake and thus increase the risk of being overweight^19^. Indeed, the results of the study showed that the frequency of cake and fast food consumption had a significant relationship with overweight and obesity. So, among overweight and obese students, 77% always or often consumed them. Other studies have shown that 76.4% of overweight and obese students consumed sweetened products and 33.3% consumed fat^12^.

The media (magazines, television and social networks) are important in changing the perception of the ideal body image. The study indicated that exposure to these media was positively related to the desire to lose weight. Our results are comparable to those of other studies in Western and Arab countries. Exposure to magazines^20^ and television^21^ was strongly associated with the desire to lose weight and therefore with dissatisfaction with body image among women. It was also reported that the more Arab women are exposed to Western models, the thinner they wanted to be. Similarly, authors^22^ have reported that Western advertising and media have induced Jordanian women to want to be thinner. In Egypt, exposure to fashion magazines and television was found to be significantly associated with women's body dissatisfaction and desire to be slimmer^23^. In general, students who frequently read women's magazines are two to six times more at risk of weight loss than those who rarely read those^24^. As for television, the risk of dieting for weight loss is higher among women and university students who are frequently exposed to fashion television channels^24^.

In terms of weight estimation, most participants were objective and our results were comparable to those reported in other studies^21^,^25^. However, there are others that have shown different results and that students tend to over- or underestimate their body weight^10^,^26^,^27^. As in other studies^28^, almost 2/3 of the students were dissatisfied with their body weight although the majority of them had a normal BMI. This proportion is twice that published by other studies^29^. This could be explained by the westernization of societal behaviors and the change in perception by preferring thinness to overweight and obesity. This dissatisfaction made them want to adopt attitudes in favor of weight loss^25^,^30^. This choice was guided by factors such as friends, family, magazines and mass media^10^,^31^,^32^.

Our study also revealed that weight perception is not related to ethnicity. Nevertheless, more than half of the Saharian students (n=15) considered overweight/obesity to be normal, while more than three-quarters of the students from other ethnic groups considered it to be a disease. This difference could be explained by the cultural diversity between Moroccan ethnic groups and the effect of economic and socio-cultural modernization. Indeed, in the Arab culture, the thin woman was considered socially undesirable, while roundness is seen as a symbol of fertility and femininity^33^. However, this perception of Arab culture has changed, as thinner women are considered more attractive^34^. In African culture, even when black women perceive themselves to be overweight; they still see themselves as physically attractive^35^.

In southern Morocco, a fat woman is not only a sign of beauty but also of richness, while women's thinness is a sign of poverty^36^. However, others studies^23^ have found that 81% of female university students in Kuwait thought that men preferred thin women.

## Study limitations

For administrative reasons and the collaboration of the responsible persons, only two institutes of higher education were selected.

The results obtained were based on the students' statements, given the difficulty of carrying out a prospective study to assess the diet and physical activity of female students. To reduce this limitation, before administering the questionnaire, participants were sensitized to the importance of their statements to the study.

## Study forces

Anthropometric measurements were made directly on the students,

Choice of institutes with limited access and welcoming students from the 3 southern regions.

One of the few studies conducted among female students in higher education.

## Conclusion

The results of the study revealed that a quarter of the participants were overweight and had behavioral risk factors such as a sedentary lifestyle and an unhealthy diet, which requires the promotion of a healthy lifestyle among our young students as well as psychological support for those dissatisfied with their body image. As well, it is advisable to conduct further studies to evaluate the lifestyle of these students, especially the dietary aspect and physical activity.
